# How sleep duration mediated childhood trauma and Internet addiction of the vocational college population in urban areas of south China

**DOI:** 10.3389/fpsyt.2022.1088172

**Published:** 2023-01-13

**Authors:** He Wang, Weijun Luo, Weikang Huang, Haishan Xiang, Siqi Chen, Wei Lin, Caiyun Chen, Yingjie Zhang, Shengbing Huang, Yueyun Wang, Peiyi Liu

**Affiliations:** ^1^Department of Healthcare, Affiliated Shenzhen Maternity and Child Healthcare Hospital, Southern Medical University, Shenzhen, Guangdong, China; ^2^Department of Traditional Chinese Medicine, People’s Hospital of Longhua, Shenzhen, Guangdong, China; ^3^Guangdong Provincial Key Laboratory of Tropical Disease Research, Department of Epidemiology, School of Public Health, Southern Medical UniversityGuangzhou, Guangdong, China; ^4^School of Public Health, Zhengzhou University, Zhengzhou, Henan, China

**Keywords:** childhood trauma, Internet addiction, sleep duration, south China, young adults

## Abstract

**Background:**

Internet Addiction is positively associated with a range of psychological risk factors such as childhood trauma and sleep disorders. However, it remains unclear if sleep duration mediates the association between childhood trauma and Internet addiction.

**Methods:**

We enrolled 14,263 students from Shenzhen Polytechnic College, China. Sleep duration, Internet addiction and childhood maltreatment were assessed in these students by self-report measures, Internet Addiction Test (IAT) and Childhood Trauma Questionnaire (CTQ), respectively. With bootstrap approach and path analysis, the mediating role of sleep duration in the association between childhood trauma and Internet addiction was analysed.

**Results:**

The Internet-addicted group exhibited a higher level of the emotional abuse (EA) score, physical abuse (PA) score, sexual abuse (SA) score, a lower level of emotional neglect (EN) score and sleep duration compared with the control group (all *p* < 0.001). The CTQ total score and subscores showed a positive correlation with IAT scores both for males (*r* = 0.199, *p* < 0.001 for the total score, *r* = 0.356, *p* < 0.001 for EA, *r* = 0.270, *p* < 0.001 for PA, *r* = 0.249, *p* < 0.001 for SA, and *r* = 0.132, *p* < 0.001 for PN) and females (*r* = 0.127, *p* < 0.001 for the total score, *r* = 0.335, *p* < 0.001 for EA, *r* = 0.187, *p* < 0.001 for PA, *r* = 0.189, *p* < 0.001 for SA, and *r* = 0.065, *p* < 0.001 for PN). The CTQ subcores were negatively related to sleep duration both for males (*r* = −0.177, *p* < 0.001 for EA, *r* = −0.180, *p* < 0.001 for PA and *r* = 0.182, *p* < 0.001 for SA) and females (*r* = −0.137, *p* < 0.001 for EA, *r* = −0.105, *p* < 0.001 for PA, and *r* = −0.182, *p* < 0.001 for SA) and sleep duration was negatively correlated with IAT scores both in males (*r* = −0.120, *p* < 0.001) and females (*r* = −0.108, *p* < 0.001). Further, the path analysis suggested that EA and SA mediated significantly to the Internet addiction when all types of childhood trauma were examined in one model (both *p* < 0.001).

**Conclusion:**

In the current study, a great proportion of students met criteria for Internet addiction. Sleep duration mediated a significant proportion of the indirect effect between EA/SA and Internet addiction. The findings may help with prevention and intervention of Internet addiction in the future. The limitation of this study was that it was a cross-sectional study and not controlling for other mental disorders. Future large-scale longitudinal studies will be needed to further clarify the relationship between childhood abuse and Internet addiction and the mediation role of sleep duration.

## 1. Introduction

Internet addiction is defined as the inability to control Internet using, leading to physical, psychological and social difficulties (1). The prevalence of Internet addiction among general population in the US and Europe ranged between 1.5 and 8% (2). The prevalence of Internet addiction among adolescent/university students fluctuates between 0.9 and 33% in previous studies from the Middle East and Asia (3). According to previous studies in China, the prevalence of Internet addiction in general population was up to 36.7% during COVID-19 (4). Excessive use of the Internet comprises problematic behavior in human interactions with information and communication technologies (5), and has become a major public health problem (6). Teenagers and young adults appear to be the most susceptible to Internet addiction (7). The rising incidence of Internet addiction was often associated with poor emotional stability and self-discipline (8). Young adults with Internet addiction may manifest short sleep duration (9) and engage in Internet use to cope with emotional distress as well as to seek pleasure, social connections and a sense of achievement (10). In particular, young adults spend significantly more time building and maintaining social interactions on the Internet than adults (11). Understanding the psychological and social factors that contribute to the severity of Internet addiction is of public health importance (12).

Along with Internet addiction, childhood abuse is one of the world’s major public health problems (13). Childhood trauma is defined as the experience of a single or multiple events by a child that is emotionally painful or distressful, which often leads to seriously lifelong damages to physical and mental health (14). Childhood abuse mainly includes emotional abuse, physical abuse, sexual abuse, emotional neglect and physical neglect. A meta-analysis consisted of 55 studies from 24 countries showed that the incidence of child sexual abuse ranged from 8 to 31% among girls and from 3 to 17% among boys (15). Two other comprehensive meta-analyses showed that the incidence of physical abuse, physical neglect and emotional neglect was 17.7, 16.3, and 18.4%, respectively (16). A study of young adults in southeastern China showed that a proportion of 18.6% of university students had self-reported childhood trauma exposures (13). Exposure to childhood trauma is thought to be a major risk factor for psychosocial dysfunction, including emotional disorders, depression and increased demands associated with Internet addiction (17). Research has shown that childhood abuse is a positive predictor of Internet addiction (18). And childhood abuse may lead to structural and functional changes in the brain and stress-responsive neurobiological systems, resulting in adverse health outcomes and behaviors on adolescents and adults (19).

Sleep is an unavoidable activity in the daily living and one of the most important factors to promote health (20). As many countries reported high rates of sleep disturbance in adolescents, sleep health of adolescent has become an increasing international public health concern (21). At present, poor sleep quality is common among high school students, with research showing that the number of adolescents with poor sleep quality was up to 35% in Scottish (22). Sleep problems are also common among high school students in Guangdong province of China, with 25.66% reporting poor sleep quality (23). Sleep problems in adolescents include early waking, frequent nightmares, sleep insufficiency, daytime sleepiness, staying up late and so on (24). Existing research has shown that maltreatment during childhood is associated with sleep disturbance in later adulthood (25). Furthermore, evidence has showed that childhood abuse could increase the risk of sleep disturbance in Chinese adolescents (26). A number of studies have shown that poor sleep is associated with increasing mental and physical health consequences, such as alcohol abuse (27), depression (28), Internet addiction (29), suicidal behavior (30), and obesity (31). Similarly, a recent study also found that students with sleep disorders spend more time watching TV or surfing the Internet (32). These findings suggest that childhood abuse may affect the incidence of Internet addiction by affecting sleep condition.

In this study, the outcome variable we intended to focus more on was Internet addiction. On the one hand, with the global epidemic of COVID-19, the incidence of Internet addiction is gradually increasing (4). On the other hand, although there were studies on childhood abuse as an independent variable and Internet addiction as a dependent variable (12, 33), few studies have employed sleep duration as a mediating variable in the relationship between the two. As a result, we intended to explore whether sleep duration, which has not been widely paid attention to, would play a mediating role between childhood abuse and Internet addiction. The present study was based on the bioecological theory of human development, which was proposed by Bronfenbrenner (34). The author believed that development of adolescents was affected by a set of nested contexts (i.e., microsystem, mesosystem, exosystem, macrosystem), which indicated that influence factors of mental health of adolescents presented in the family, school, and community could be both part of the past and the present environments. Based on the above theory, this large-scale cross-sectional study would concentrate on the relationship between childhood maltreatment and Internet addiction and further explore the mediating role of sleep duration between the two. We assessed childhood trauma with the Childhood Trauma Questionnaire (CTQ) and Internet addiction with Internet Addiction Test (IAT) in both Internet-addicted and control participants. We employed mediation and path analysis to evaluate how sleep duration mediate the relationship between childhood trauma and Internet addiction.

## 2. Materials and methods

### 2.1. Study population

This study was a cross-sectional study, which recruited the students in Shenzhen Polytechnic College from May to August in 2019. A total of 14,263 subjects aged 15∼29 years old were enrolled and finished the questionnaire. The students who were on leave or sick were excluded. Subjects scanned a QR code to fill out an electronic questionnaire. All participants were asked to answer the questionnaires independently and not discuss with others. After the questionnaires were collected, the questionnaires with obvious logic errors were excluded. Finally, 13,454 valid questionnaires were obtained. The effective response rate of the questionnaire was 94.91%. This study was approved by the Ethics Committee of Shenzhen Maternity & Child Healthcare Hospital (SFYLS [2020]031).

### 2.2. Measures of childhood trauma, Internet addiction, and sleep duration

The questionnaire consists of two scales and a number of questions, including general information, sleep duration, Childhood Trauma Questionnaire Short Form (CTQ-SF) and Internet Addiction Test (IAT).

Childhood trauma was measured by the Chinese version of the CTQ-SF (35). The 28-item scale included five dimensions including physical abuse, emotional abuse, sexual abuse, physical neglect and emotional neglect. Each item was scored with five points (1 = never, 2 = occasional, 3 = sometimes, 4 = frequent, 5 = always). Some of the items include: “No one at home cared about my hunger,” “I was hit so hard that it attracted the attention of people like teachers, neighbors or doctors” and so on. The cut-off scores identifying respondents from low to severe exposure for each subscale were: emotional abuse = 13, physical abuse = 10, sexual abuse = 8, emotional neglect = 15, and physical neglect = 10 (36). Chinese version of CTQ-SF had good reliability and validity among Chinese undergraduates (35). In this study, the internal consistency of the questionnaire was good, and the Cronbach’s α coefficient of the scale was 0.763. The validity of the scale was also good (Kaiser-Meyer-Olkin coefficient = 0.934, Bartlett’s sphericity test for significance *p* < 0.001).

The IAT is a 20-item scale that measures addictive Internet use developed by Young (37), who reported a Cronbach’s alpha that ranged from 0.54 to 0.82. It has six dimensions: salience, excessive use, neglect of work, anticipation, lack of control, and neglect of social life (38). It is measured with a six-point Likert scale ranging from 0 (not at all) to 5 (always), to assess the extent to which Internet use affected daily life. Some of the items include: “How often do you fear that life without the internet would be boring, empty, and joyless,” “How often do you lose sleep over logging on to your computer late at night.” A higher score indicates a higher degree of Internet addiction: normal: 0∼39; mild: 40∼59; moderate: 60∼79; and severe: 80∼100. This measure has been widely used around the world in studies investigating Internet addiction and has been validated in many populations. A more recent review and meta-analysis study suggested that the IAT had an acceptable internal consistency, test-retest reliability and convergent validity among a total of 25 studies including 18,421 subjects (39). Based on previous studies, we employed a cut-off score of = 40 to identify individuals with Internet addiction according to Young’s criteria (40, 41). The current study population comprised 5,089 (2,193 males and 2,896 females) Internet-addicted individuals and 7,236 (3,114 males and 4,122 females) non-Internet-addicted individuals. In this study, the Kaiser-Meyer-Olkin coefficient = 0.952, Bartlett’s sphericity test for significance *p* < 0.001, and Cronbach’s α was 0.922, indicating a high reliability and validity.

Sleep duration were obtained by self-report in the questionnaire (42). The question about sleep duration was a fill-in-the-blank question below: In the last month, how many hours and minutes of sleep did you have per day in average?

### 2.3. Statistical analysis

Epidata 3.1 was used to input the questionnaire information. SPSS 26.0 software was used to analyze the data. The data cleaning method in statistics was used to filter out invalid values and missing values, and we check the consistency of data to ensure the validity and accuracy. We conducted an χ^2^ test to analyze the grade and household variables and a Student’s *t*-test for other variables. A Spearman’s correction was performed to analyze the relationships between CTQ scores, sleep duration and IAT scores. Two-sided *p*-values less than 0.05 were considered to be statistically significant in this study.

Mediation analysis was applied to further explore whether sleep duration played a mediation role in the association of childhood trauma and Internet addiction by using the Bootstrap method and the SPSS PROCESS 4.0 package, and Bootstrap was set to 10,000 times. The test level was α = 0.05. In the mediation analysis, the relation between the independent variable CTQ score and the dependent variable IAT score was tested to see if it was significantly mediated by sleep duration. Path a. the association between CTQ score and sleep duration (the mediator). Path b. the association between sleep duration and IAT score (the outcome). Path c and Path c’, the total and direct effects of CTQ score on IAT score, respectively. Full mediation effect exists when the indirect effect was significant.

Using AMOS 26.0, we performed path analysis to identify the relationships between childhood trauma, sleep duration and Internet addiction by structural equation modeling (SEM). The advantage of SEM is that it can simultaneously estimate multiple interdependent relationships, identify the potential relationships of these variables, and obtain the direct and indirect effects. Importantly, SEM can deal with multiple dependent variables simultaneously. Since each CTQ subfraction was highly correlated, sleep duration, IAT and all CTQ subfractions were incorporated into one model by SEM to illustrate the correlation between each CTQ subfraction. SEM has been widely used in the research of Internet addiction.

## 3. Results

### 3.1. Sociodemographic characteristics and clinical variables

The prevalence of Internet addiction in this sample was 41.29% (5,089/12,325) based on Young’s criteria of an IAT score = 40. Of the 5,089 individuals in the Internet-addicted group, 43.09% (2,193/5,089) were males and 56.85% (2,896/5,089) were females. The mean ± standard deviation values of the clinical variables, the statistics of the group main effect, and sex main effect were shown in Table 1. Male students had higher BMI than female students (*p* < 0.001), and there was no statistical difference in BMI between the Internet addiction group and the non-Internet addiction group. The frequency of weekly physical exercise of male students was higher than that of female students (*p* < 0.001), and the frequency of weekly physical exercise of Internet addiction group was lower than that of non-Internet addiction group (*p* < 0.001). The Internet-addicted vs. the control group exhibited a higher level of the IAT score, EA score, PA score, SA score (all *p* < 0.001) and a lower level of EN score and sleep duration (all *p* < 0.001) but not CTQ total score (*p* = 0.063) and PN (*p* = 0.077) score. In the sex main effect, the females showed a higher level of IAT score, CTQ total score, EN score, PN score, sleep duration (all *p* < 0.001) and a lower level of PA and SA score (all *p* < 0.001) but not EA score (*p* = 0.537) compared with males.

**TABLE 1:** Sociodemographic characteristics and clinical variables of the participants.

Characteristic	Male	Female IA (*n* = 2896)	Male control	Female control	Group	Sex
	IA (*n* = 2193)		(*n* = 3114)	(*n* = 4122)		
Age	19.88 ± 2.07	19.61 ± 1.15	19.93 ± 1.99	19.66 ± 1.09	F = 2.21	F = 3.62
					*p* = 0.137	*p* = 0.057
BMI	21.31 ± 3.74	20.73 ± 5.29	21.43 ± 3.61	20.55 ± 5.17	F = 0.27	F = 120.31
					*p* = 0.601	*p* < 0.001***
Grade	–	–	–	–	χ^2^ = 21.65	χ^2^ = 56.39
					*p* < 0.001***	*p* < 0.001***
1	426 (19.4%)	543 (18.7%)	544 (17.5%)	687 (16.7%)	–	–
2	978 (44.6%)	1,441 (49.7%)	1,307 (42.0%)	2,023 (49.1%)	–	–
3	782 (35.7%)	909 (31.4%)	1,257 (40.4%)	1,410 (34.2%)	–	–
4	7(0.3%)	4(0.1%)	6(0.2%)	2(0)	–	–
Physical activity (times/week)	2.19 ± 1.97	1.76 ± 1.82	2.78 ± 2.18	2.02 ± 1.89	F = 64.22	F = 150.95
					*p* < 0.001***	*p* < 0.001***
Household	–	–	–	–	χ^2^ = 37.63	χ^2^ = 126.82
					*p* < 0.001***	*p* < 0.001***
Country	1,015 (46.3%)	1,523 (52.6%)	1,148 (36.9%)	2,055 (49.9%)	–	–
City	1,178 (53.7%)	1,374 (47.4%)	1,966 (63.1%)	2,067 (50.1%)	–	–
IAT	49.97 ± 10.85	49.48 ± 9.28	29.20 ± 6.17	30.50 ± 5.53	F = 941.24	F = 0.003
					*p* < 0.001***	*p* = 0.956
Sleep duration	6.60 ± 1.44	6.76 ± 1.15	6.96 ± 1.41	6.98 ± 1.14	F = 47.58	F = 51.82
					*p* < 0.001***	*p* < 0.001***
CTQ	55.81 ± 11.69	56.32 ± 8.45	53.62 ± 10.86	55.18 ± 8.48	F = 3.46	F = 244.80
					*p* = 0.063	*p* < 0.001***
EA	7.58 ± 3.14	7.77 ± 3.08	6.03 ± 1.94	6.35 ± 2.15	F = 833.62	F = 0.38
					*p* < 0.001***	*p* = 0.537
PA	6.66 ± 2.94	5.92 ± 2.07	5.59 ± 1.68	5.42 ± 1.38	F = 876.37	F = 285.88
					*p* < 0.001***	*p* < 0.001***
SA	6.02 ± 2.66	5.46 ± 1.51	5.23 ± 1.12	5.15 ± 0.85	F = 1044.94	F = 320.23
					*p* < 0.001***	*p* < 0.001***
EN	17.02 ± 5.50	18.07 ± 5.11	18.44 ± 6.11	19.28 ± 5.25	F = 11.60	F = 118.50
					*p* < 0.001***	*p* < 0.001***
PN	11.54 ± 2.96	11.77 ± 2.39	11.19 ± 2.94	11.60 ± 4.40	F = 3.12	F = 230.04
					*p* = 0.077	*p* < 0.001***

Values are mean ± SD or the actual number (percentage%), IAT, Internet addiction test; CTQ, childhood trauma questionnaire; EA, emotional abuse; PA, physical abuse; SA, sexual abuse; EN, emotional neglect; PN, physical neglect.

***Indicate significance at *p* < 0.001.

### 3.2. Associations between the PA, IAT, and CTQ scores in the Internet-addicted group

Spearman’s rank correlation coefficient was used to analyze the association between the IAT score, sleep duration, CTQ total score and all subscores for the Internet-addicted group (Table 2). The CTQ total score and subscores showed a positive correlation with IAT score both for males (*r* = 0.199, *p* < 0.001 for the total score, *r* = 0.356, *p* < 0.001 for EA, *r* = 0.270, *p* < 0.001 for PA, *r* = 0.249, *p* < 0.001 for SA, and *r* = 0.132, *p* < 0.001 for PN) and females (*r* = 0.127, *p* < 0.001 for the total score, *r* = 0.335, *p* < 0.001 for EA, *r* = 0.187, *p* < 0.001 for PA, *r* = 0.189, *p* < 0.001 for SA, and *r* = 0.065, *p* < 0.001 for PN). The CTQ subscores were negatively related to sleep duration both for males (*r* = −0.177, *p* < 0.001 for EA, *r* = −0.180, *p* < 0.001 for PA and *r* = 0.182, *p* < 0.001 for SA) and females (*r* = −0.137, *p* < 0.001 for EA, *r* = −0.105, *p* < 0.001 for PA, and *r* = −0.182, *p* < 0.001 for SA). Further, the sleep duration was negatively correlated with IAT score both in males (*r* = −0.120, *p* < 0.001) and females (*r* = −0.108, *p* < 0.001).

**TABLE 2:** Spearman’s rank correlation coefficients between childhood trauma, sleep duration, and Internet addiction.

	IAT	CTQ	EA	PA	SA	EN	PN
**Male**							
Sleep duration	*r* = −0.120**	*r* = 0.054**	*r* = −0.177**	*r* = −0.180**	*r* = −0.182**	*r* = 0.214**	*r* = 0.290**
	*p* < 0.001	*p* < 0.001	*p* < 0.001	*p* < 0.001	*p* < 0.001	*p* < 0.001	*p* < 0.001
IAT	/	*r* = 0.199**	*r* = 0.356**	*r* = 0.270**	*r* = 0.249**	*r* = −0.049**	*r* = 0.132**
		*p* < 0.001	*p* < 0.001	*p* < 0.001	*p* < 0.001	*p* < 0.001	*p* < 0.001
**Female**							
Sleep duration	*r* = −0.108**	*r* = 0.067**	*r* = −0.137**	*r* = −0.105**	*r* = −0.104**	*r* = 0.164**	*r* = 0.058**
	*p* < 0.001	*p* < 0.001	*p* < 0.001	*p* < 0.001	*p* < 0.001	*p* < 0.001	*p* < 0.001
IAT	/	*r* = 0.127**	*r* = 0.335**	*r* = 0.187**	*r* = 0.189**	*r* = −0.100**	*r* = 0.065**
		*p* < 0.001	*p* < 0.001	*p* < 0.001	*p* < 0.001	*p* < 0.001	*p* < 0.001

IAT, Internet addiction test; CTQ, childhood trauma questionnaire; EA, emotional abuse; PA, physical abuse; SA, sexual abuse; EN, emotional neglect; PN, physical neglect.

**Indicate significance at *p* < 0.01.

### 3.3. Mediation and path analyses for Internet-addicted individuals

**FIGURE 1:**
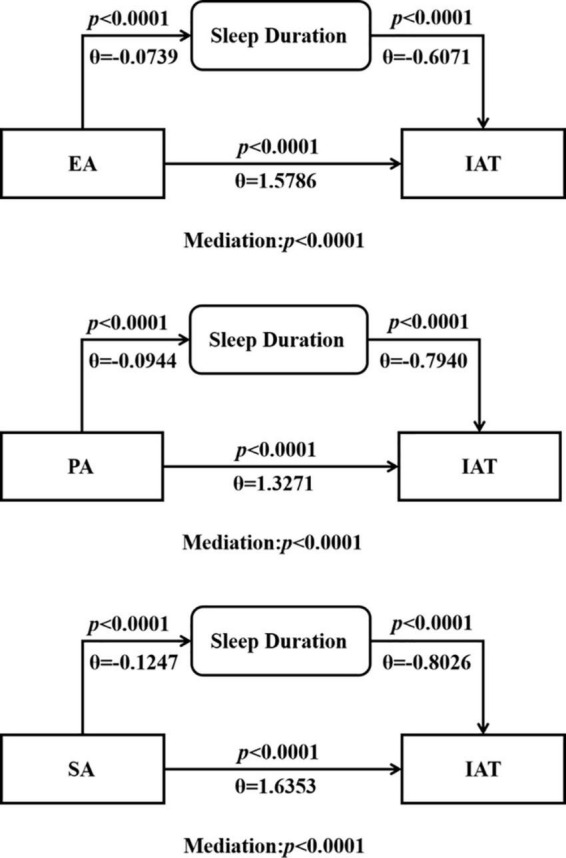
Mediation analysis of the sleep duration, IAT and childhood trauma questionnaire (CTQ) subscores in Internet-addicted group. IAT, Internet addiction test; EA, emotional abuse; PA, physical abuse; SA, sexual abuse. The *p*-values associated with mediation are for the path c–c’. All models are significant, suggesting that the sleep duration mediated the relationship between the CTQ subscores and the IAT score.

As the CTQ subscore (EA, PA, and SA score) and sleep duration were pair-wise correlated with Internet addiction, a mediation analysis was conducted to examine whether the sleep duration mediated the relationship between the CTQ EA, PA and SA subscore and the IAT score. Findings from the mediational analysis with sleep duration as the mediator is presented in Table 3. Regression analysis showed that childhood abuse (EA, PA, SA score) had a significant positive predictive effect on Internet addiction (*b* = 1.579, 1.327, 1.635, *p* < 0.01). When sleep duration was included in the regression equation, the indirect effects of EA, PA, and SA on Internet addiction mediated by sleep duration were 0.045, 0.075, and 0.100, and the corresponding contribution rates of sleep duration were 2.8, 5.7, and 6.1%, respectively. Figure 1 showed that childhood abuse (EA, PA, and SA score) negatively predicted sleep duration (*b* = −0.074, −0.094, −0.125, *p* < 0.01), and sleep duration negatively predicted Internet addiction (*b* = −0.607, −0.794, −0.803, *p* < 0.01). The result showed that all models were significant with the sleep duration mediating the correlation between the CTQ subscore (EA, PA, and SA score) and IAT score.

**TABLE 3:** Effect estimates of effects of childhood trauma on Internet addiction mediated *via* sleep duration.

	EA		PA		SA	
	*b*	95% CI	*b*	95% CI	*b*	95% CI
Indirect effect	0.045**	(0.028, 0.063)	0.075**	(0.051, 0.102)	0.100**	(0.069, 0.135)
Direct effect	1.579**	(1.500, 1.657)	1.327**	(1.220, 1.434)	1.635**	(1.497, 1.774)
Total effect	1.623**	(1.545, 1.702)	1.402**	(1.296, 1.508)	1.735**	(1.598, 1.873)
Proportion of total effect mediated	0.028	–	0.053	–	0.058	–
Ratio of indirect to direct effect	0.028	–	0.057	–	0.061	–

EA, emotional abuse; PA, physical abuse; SA, sexual abuse. *b*, unstandardized coefficient; 95% CI, 95% confidence interval.

**Indicate significance at *p* < 0.01.

**FIGURE 2:**
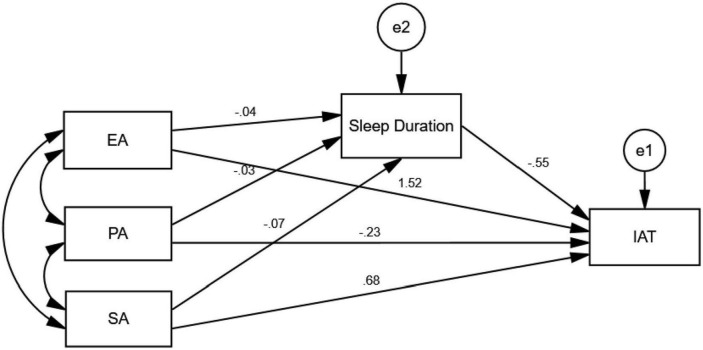
Path analysis of childhood trauma questionnaire (CTQ subscores), the sleep duration and Internet addiction (IAT score) for Internet-addicted group. IAT, Internet addiction test; EA, emotional abuse, PA, physical abuse; SA, sexual abuse. e1 and e2 represent the error terms of the sleep duration and IAT scores. The value on each path indicates the standardized coefficient, and the *p*-values in the figure were all <0.0001.

A path analysis was applied to include the sleep duration, IAT score and three CTQ subscores (EA, PA, SA score) in one model for Internet-addicted group (Figure 2). The model showed that EA (β = 1.52, *p* < 0.001) and SA (β = 0.68, *p* < 0.001) had significant positive effects on the IAT. The EA (β = −0.04, *p* < 0.001), PA (β = −0.03, *p* < 0.001) and SA (β = −0.07, *p* < 0.001) score were negatively related to sleep duration, and the sleep duration was negatively correlated to IAT score (β = −0.55, *p* < 0.001). These results suggested that sleep duration contributed a significant partial mediating role between childhood abuse (EA and SA score) and Internet addiction.

## 4. Discussion

In this study, we assessed the relationships between childhood trauma, sleep duration and Internet addiction in Internet-addicted college students in urban areas of south China, and we found childhood trauma total score was positively associated with Internet addiction and the sleep duration, while the sleep duration showed a negative relationship with Internet addiction. Differently, three CTQ subscores (emotional abuse, physical abuse, and sexual abuse) were positive related to Internet addiction, and had a negative relationship with sleep duration. Another important finding is that sleep duration mediated the relationships between childhood trauma (emotional abuse, physical abuse, and sexual abuse) and Internet addiction. A path analysis further suggested that emotional abuse and sexual abuse contributed more significantly to Internet addiction when three childhood trauma subtypes (emotional abuse, physical abuse, and sexual abuse) were examined together in one model.

### 4.1. Childhood trauma and Internet addiction

In the past decade, Internet addiction of young people has gradually become a worldwide psychological behavior problem (6). According to domestic meta-analytic studies, the prevalence rate of Internet addiction was between 11.3% (43) and 30.1% (44). Internet addiction has a wide range of harms to young people, which may lead to damage to their physical, psychological, emotional and social functions (45), such as social dysfunction, frequent family conflicts and inability to complete their studies (46), which may result in substantial consequences of school failure and social cost (47). Therefore, measures should be taken to intervene the phenomenon of Internet addiction among adolescents and young adults. Existing studies have found that traumatic experience in childhood is significantly correlated with adolescent problem behaviors (48), which might be an important factor in the pathogenesis of Internet addiction. This study showed that Internet addiction was a serious problem in vocational college population. We found that the detection rate of Internet addiction among vocational college students in Shenzhen was 41.29%, which was higher than other studies of the same kind (12). There was no significant difference in the prevalence of Internet addiction between male and female students, which was similar to other studies (12). This might be related to the high penetration of Internet and smart phones in recent years, the equal access to electronic products and the opportunities for male and female students to use the Internet. At the same time, the total score of childhood trauma questionnaire and four sub-dimensions (emotional abuse, physical abuse, sexual abuse and physical neglect) had a significant predictive effect on Internet addiction, which was consistent with previous studies. This finding suggests that Internet addiction among young people originated early during their life courses, and childhood trauma may predispose young people at higher risk of Internet addiction. Childhood trauma can lead to catastrophic effects on individuals’ physical, emotional, cognitive and social development (49). Early adverse experiences could damage brain functions by affecting the development process of the brain, and the potential mechanism(s) are stress response-related overactivation of the hippocampal-amygdalar complex coupled with N-methyl-D-aspartate (NMDA) receptor-mediated neurotoxicity driven by stress response-related elevations of glucocorticoids (50), resulting in abnormal neurobiological pathways, impulse control and executive dysfunction (46). It has also been suggested that behavioral addiction can be considered a manifestation of an individual’s underlying psychopathological vulnerability, such as impulsivity, anhedonia, dissociative symptoms and alexithymia, rather than a symptom of excessive involvement in maladaptive activities *per se* (51). The result of the research confirmed that there might be risk factors formed from early life for Internet addiction behavior from the psychological perspective. In China, there is an old saying that beating is a sign of intimacy and scolding shows love. Childhood abuse was often a recurring behavior, therefore children who were treated this way were more likely to develop Internet addiction in their adulthood. To develop effective intervention programs of Internet addiction for adolescents and young adults, we can start from childhood health education for parents, and gradually change the education concept of the older generation of parents and further their own behavior.

### 4.2. The mediating role of sleep duration in the relationship between childhood trauma and Internet addiction

A number of studies found that the incidence of sleep problems was 8.7–59.5% in people who had suffered childhood maltreatment (52), and childhood maltreatment could positively predicted high sleep disorders and low sleep quality in adolescents (26). The relationship between childhood abuse and sleep disorders has been confirmed in a cohort study (52). However, most of the current studies on sleep disorders and childhood trauma were cross-sectional studies, therefore it was difficult to verify the causal relationship between the two. This study also showed that there was a negative correlation between childhood maltreatment (emotional abuse, physical abuse and sexual abuse) and sleep duration both in males and females, which was consistent with previous research. Childhood abuse is an extremely important type of early life stress (53). It can activate the hypothalamic–pituitary–adrenal (HPA) system leading to cardiovascular, catecholamine, cortisol, adrenocorticotropic hormone (ACTH), and corticotropin-releasing hormone (CRH) hyperactivity (54). Increasing number of researches manifest that there is a correlation between sleep and HPA axis activity (55). In addition, there is evidence that hyperactivity of HPA axis had many negative effects on sleep, including sleep fragmentation, reduced slow-wave sleep (SWS), and shorter sleep duration (56), which is consistent with our study. The evidence for the role of CRH in regulating sleep was also well-established (57). In addition, prolonged central CRH secretion is thought to be associated with sleep disorders (58). Therefore, we propose that childhood maltreatment might increase HPA axis activity, which enhances CRH secretion (59) and ultimately leads to an increased risk of shortened sleep duration (60). An earlier study showed that students with sleep problems spent more time watching television and surfing the social networking websites (29), and these students were more likely to develop Internet addiction. Our study also found a negative correlation between sleep duration and Internet addiction. In our study, sleep duration independently mediated 2.8–6.1% of the indirect effect of childhood trauma on Internet addiction. This finding suggested that it was not entirely childhood trauma or less sleep duration *per se* that increased risk of Internet addiction but a battery of predictors including childhood trauma and sleep duration that affected Internet addiction. A recent study suggested that a wide range of abnormal connections in the hippocampus might mediate sleep disorders and Internet addiction, and sleep disorders may further aggravate the degree of IAT and be mediated by this connectivity (61). On the other hand, based on Bronfenbrenner’s bioecological theory of human development (34), individual behavior is not only directly influenced by life events in the social environment, but also indirectly influenced by events in the larger community, nationwide, and even worldwide. In his theoretical model, Bronfenbrenner called the environment in which people live and the interaction with the environment as “behavioral system” and divided the system into four levels from small to large, namely, microcosmic system, intermediate system, outer system, and macro system. Parents’ behaviors directly affect children’s growth and development in the microcosmic system. Therefore, sleep disorder and Internet addiction might be emotional responses to childhood trauma (62). It is worth mentioning that this study was a large-scale study on the psychological status of middle and high school students. Considering that the length of the questionnaire was a factor affecting the quality of the study, we tried to simplify the questionnaire in compliance with the scientific and operational premise when designing the questionnaire. A review of the literature (42) revealed that sleep duration was a relatively intuitive metric, and similar literature had used sleep duration to assess sleep status (63). As a result, we used sleep duration as a measure of sleep. In future large-scale studies, we will adopt internationally used sleep assessment scales, such as Pittsburgh Sleep Quality Assessment Scale, to strengthen the validity and comprehensive performance of sleep quality measurement, so as to verify the mediating role of sleep quality in childhood abuse and Internet addiction.

The findings from this study indicate that the experience of childhood maltreatment predicts shorter sleep duration in adolescents and young adults. Therefore, our findings were useful for identifying adolescents and young adults who might be at risk of shorter sleep duration and at high risk of Internet addiction. In addition, these findings highlighted the importance of accessing childhood maltreatment history, especially in adolescents and young adults with shorter sleep duration. Furthermore, understanding the mediating role of sleep duration between childhood maltreatment and Internet addiction might also help the prevention and treatment interventions of shorter sleep duration for adolescents and young adults who suffered from childhood maltreatment. Finally, we suggested that childhood maltreatment should be prevented from happening in the first place (64). Additionally, as the evidence links reduced sleep duration to Internet addiction, adolescents and young adults with shorter sleep duration should receive more comprehensive intervention and long-term treatment from clinicians (65).

### 4.3. Strengths, limitations, and conclusion

The strengths of our study are as follows. First, we performed a comprehensive analysis of the association between sleep duration, childhood trauma, and Internet addiction in a multi-factor model. Besides, we conducted path analysis which is a new approach to assess the potential role of sleep duration in the associations between childhood trauma and Internet addiction.

However, several limitations of our study should be noted. First, the current results were based on a cross-sectional study. We are unable to determine the causal relationships between childhood trauma and Internet addiction. Therefore, this pathway should be validated in larger cohort studies. Secondly, the sleep duration of participants in this study was obtained by means of self-report rather than validated questionnaires. There may be some recall bias and misclassification. Furthermore, the associations between childhood trauma and Internet addiction were also affected by other mental disorders, which were not assessed in our analysis. Future large-scale longitudinal studies may be able to include screening for relevant psychiatric symptoms to further clarify the relationship between childhood abuse and Internet addiction.

In conclusion, this study provided important evidence that childhood trauma was negatively associated with sleep duration, and sleep duration was negatively related to Internet addiction in young Chinese adults. Sleep duration played an important role in the association between childhood trauma and Internet addiction. It is important to understand the mediating role of sleep duration between child abuse and Internet addiction, which will provide new ideas and strategy for solving the problem of Internet addiction from public health prospective. It is equally important that these findings need to be confirmed in future multi-center, large-scale studies.

## Data availability statement

The raw data supporting the conclusions of this article will be made available by the authors, without undue reservation.

## Ethics statement

The studies involving human participants were reviewed and approved by Ethics Committee of Shenzhen Maternity and Child Healthcare Hospital. Written informed consent from the participants’ legal guardian/next of kin was not required to participate in this study in accordance with the national legislation and the institutional requirements.

## Author contributions

YW: conception and design of study. HW, CC, YZ, and SH: acquisition of data. HW, HX, SC, and WLi: analysis of data. HW, PL, WLu, and WH: revising the manuscript critically for important intellectual content. HW: drafting the manuscript. All authors contributed to the article and approved the submitted version.
